# Screening colorectal tumor-associated fibroblasts using dual-channel THz biosensor

**DOI:** 10.1016/j.isci.2026.117735

**Published:** 2026-10-19

**Authors:** Hongji Xu, Ruiqi Gu, Arthur Georg Serville Torvund, Iver Grønlien, Angelos Xomalis, Hai Son Nguyen, Zhengping Yu, Zhenyu Zhao

**Affiliations:** 1 Department of Physics, Shanghai Normal University, 100 Guilin Road, Shanghai 200234, China; 2 Clinical Department of Colorectal Surgery, Fudan University Shanghai Cancer Center, Shanghai 200032, China; 3 Nanoelectronics and Photonics Group, Department of Electronic Systems, Norwegian University of Science and Technology, 7491 Trondheim, Norway; 4 CNRS-International-NTU-Thales Research Alliance (CINTRA), IRL 3288, Singapore 637553, Singapore; 5 Ecole Centrale de Lyon, INSA Lyon, Université Claude Bernard Lyon 1, CPE Lyon, CNRS, INL, 9 UMR5270, 69130 Ecully, France

**Keywords:** metasurface, quasi-bound states in the continuum, electromagnetically induced transparency, bio-sensors, colorectal tumor-associated fibroblasts

## Abstract

In clinical oncology, label-free identification of colorectal tumor-associated fibroblast (CAF) subtypes is challenging. In this work, a terahertz (THz) metasurface exhibiting dual channels of probation, quasi-bound states in the continuum (q-BICs) and electromagnetically induced transparency (EIT), is used to screen CAF subtypes in colorectal tumors. When impinging light is polarized along *x* axis, the pro-tumorigenic myofibroblasts (myCAFs) cause frequency red-shift of q-BIC resonances compared to intestinal epithelial cells, while anti-tumorigenic antigen-presenting CAFs (apCAFs) result in blueshift. Similarly, for EIT modes, when light is polarized along the orthogonal polarization, myCAFs result in a frequency red-shift while apCAFs result in blueshift. These frequency shifts are attributed to the dielectric environment variation due to the surface hall-marks of CAFs. To validate our results, we conducted flow cytometry and gene expression analyses that confirmed distinct hallmarks of myCAFs and apCAFs, consistent with the resonance frequency shifts observed in THz metasurface measurements. Our findings demonstrate a label-free and cost-effective method for screening CAF subtypes using two-parameters—q-BICs and EIT—which can help speed up cancer diagnosis.

## Highlights

• Terahertz metasurface quasi-bound states in the continuum sense tumor fibroblasts
• Electromagnetically induced transparency adds a second, orthogonal sensing channel
• Myofibroblasts red-shift both resonances, antigen-presenting fibroblasts blueshift
• Flow cytometry and gene expression confirm the markers behind the terahertz readout

## Graphical abstract

**Figure:**
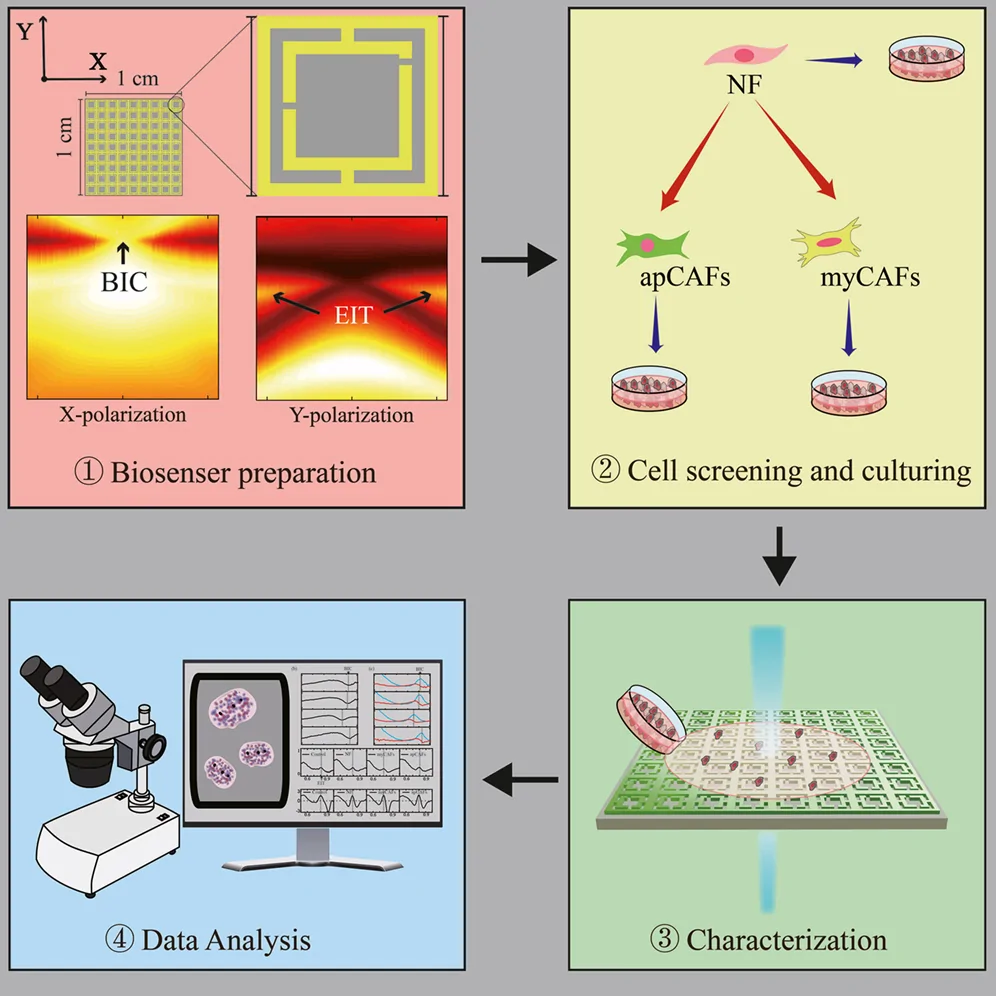

## Teaser

Screening in health technology; Radiation physics; Applied sciences

## Introduction

Colorectal cancer (CRC) is a malignant tumor originating from the colonic mucosa,^1^^,^^2^^,^^3^ leading to million deaths annually due to metastasis.^4^ Tumor metastasis is a complex, multi-factorial, and multi-stage process influenced by the tumor microenvironment (TME).^5^ Cancer-associated fibroblasts (CAFs), which comprises up to 70% of the TME,^6^ playing a crucial role in restructuring the extracellular matrix, promoting epithelial-to-mesenchymal transition (EMT), and interacting with cancer cells or other stromal cells through the secretion of cytokines, chemokines, growth factors, and extracellular components,^7^ thereby influencing tumor cell metastasis. Due to substantial evidence showing that CAFs contribute to cancer progression, targeting CAFs has emerged as a unique opportunity to control cancer. Different subtypes of CAFs exhibit totally different biological effects on tumors growth, such as immune-suppression or immune enhancement.^8^ The function and subtype of CAFs as well as the relation to the CRC are shown in Figure 1. Currently, both flow cytometry and gene expression profiling^9^^,^^10^^,^^11^ are the major tools to identify the subtype of CAFs by monitoring the hallmarks of CAFs, which is time-consuming, is expensive, and lacks identification standards. As such, a label-free and accurate method to identifying the subtypes of CAFs is essential for the clinical oncology of CRC.

**Figure 1:**
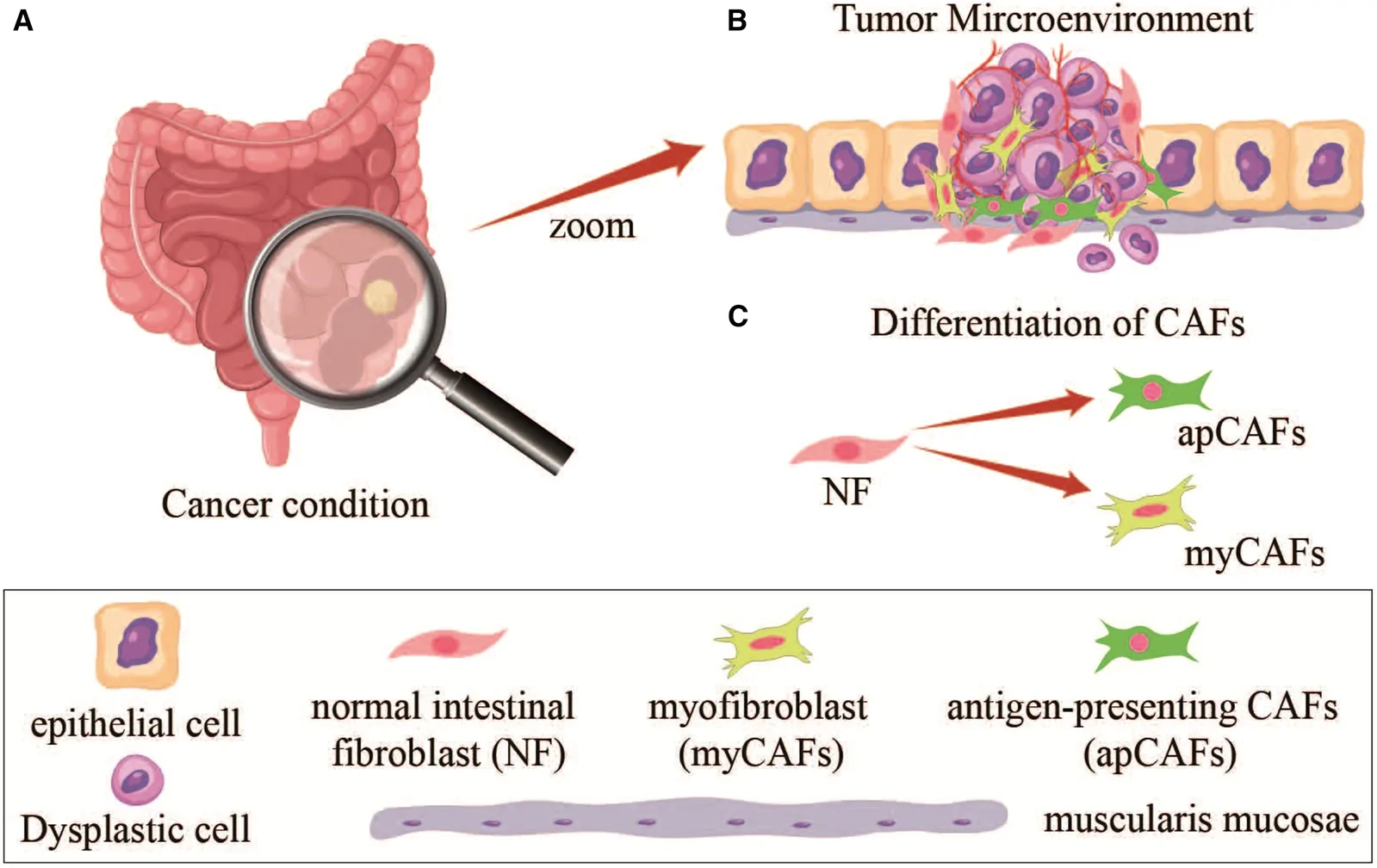
Colorectal tumor-associated fibroblasts and their microenvironment (A) CRC tumor formation. (B) Schematic representation of the major tumor microenvironment. (C) Heterogeneous differentiation of normal intestinal fibroblasts into distinct CAF subtypes. The inset shows the main parts/components of the tumor microenvironment.

Terahertz (THz) metasurfaces are emerging as label-free biosensor components for cancer detection.^12^^,^^13^^,^^14^^,^^15^^,^^16^ Designing optically resonant building blocks in the metasurface (referred to as “meta-atoms”), it is possible to modify and enhance the interactions between long wavelengths and low energy mid-infrared and THz waves and analytes in their proximity, including molecules, viruses, and proteins, thereby significantly amplifying their fingerprints within this THz range.^17^^,^^18^^,^^19^^,^^20^^,^^21^ Such metasurfaces used as biosensors facilitate strong optical resonances, such as electromagnetically induced transparency (EIT)^22^^,^^23^^,^^24^ and quasi-bound states in the continuum (q-BICs).^25^^,^^26^^,^^27^^,^^28^^,^^29^^,^^30^^,^^31^^,^^32^^,^^33^^,^^34^^,^^35^^,^^36^ One can achieve precise control over the electromagnetic waves parameters such as amplitude, polarization, phase, propagation mode, and polarization^37^^,^^38^^,^^39^ by optimizing the meta-atoms dimensional parameters, such as periodic arrangements, geometric shapes, and material compositions.^31^^,^^32^ This enables to improve the performance of natural materials, leading to applications in biomedical fields such as health care, disease diagnosis, and drug development.^31^ However, most of the THz biosensor detects the subtle frequency shift either by q-BICs or EIT.^12^^,^^13^^,^^14^^,^^15^^,^^16^^,^^17^^,^^18^^,^^19^^,^^20^^,^^21^^,^^22^^,^^23^^,^^24^^,^^25^^,^^26^^,^^27^^,^^28^^,^^29^^,^^30^^,^^31^^,^^32^^,^^33^^,^^34^^,^^35^^,^^36^^,^^37^^,^^38^^,^^39^^,^^40^^,^^41^ Achieving q-BICs and EIT simultaneously within a single metasurface could greatly increase the sensitivity of biological detection drastically.

In this work, we present a label-free approach for screening CAF subtypes utilizing a THz metasurface that features sharp q-BIC resonance of at 0.92 THz and EIT at 0.82 THz when illuminated with *x*- and *y*-polarized THz wave, respectively. By recording the responses of the metasurfaces functionalized with different subtypes CAFs through THz time-domain spectroscopy (THz-TDS), we observe that myCAFs induces a pronounced frequency red-shift while the apCAFs cause a frequency blueshift for both q-BIC and EIT modes, compared to the intestinal epithelial cells. These frequency shifts are attributed to the unique dielectric environments created by the surface markers of each CAF subtype. Furthermore, the flow cytometry and gene expression measurements reveal surface hall-markers of CAFs, which are in great agreement with our findings of THz biosensing. Collectively, these results demonstrate that our dual-parameter (q-BIC and EIT) metasurface biosensor provides a rapid, cost-effective, and label-free method for differentiating CAF subtypes, offering a promising tool for accelerating cancer diagnosis and advancing clinical oncology applications.

## Results and discussion

The THz biosensor is based on a metasurface of contact-coupled asymmetric double-arm split-ring resonators (ADSRR), whose unit cell is shown in Figure 2A. As illustrated by the simulated transmission maps in Figures 2B and 2C, the metasurface supports a q-BIC resonance near 0.92 THz under x-polarized excitation (Ex) and an EIT window near 0.82 THz under y-polarized excitation (Ey). The corresponding field and surface-current distributions (Figures 2D and 2E) reveal that the q-BIC arises from the interference between a magnetic dipole and four L-shaped electric dipoles, whereas the EIT originates from the destructive interference of two oppositely circulating magnetic dipoles. The asymmetry parameter d = 20.5 μm was selected to balance the resonance amplitude, Q factor, and FoM of both modes (Figures 2F and 2G). Three fibroblast subtypes (NF, myCAFs, and apCAFs) were then sorted and immobilized onto identical chemically functionalized metasurfaces; their morphologies before and after deposition are shown in Figures 3A and 3B, respectively. The functionalized biosensors were finally characterized by THz-TDS, with the incident pulse propagating along the z-axis normal to the metasurface, as schematically illustrated in Figure 4A (see STAR Methods for details).

**Figure 2:**
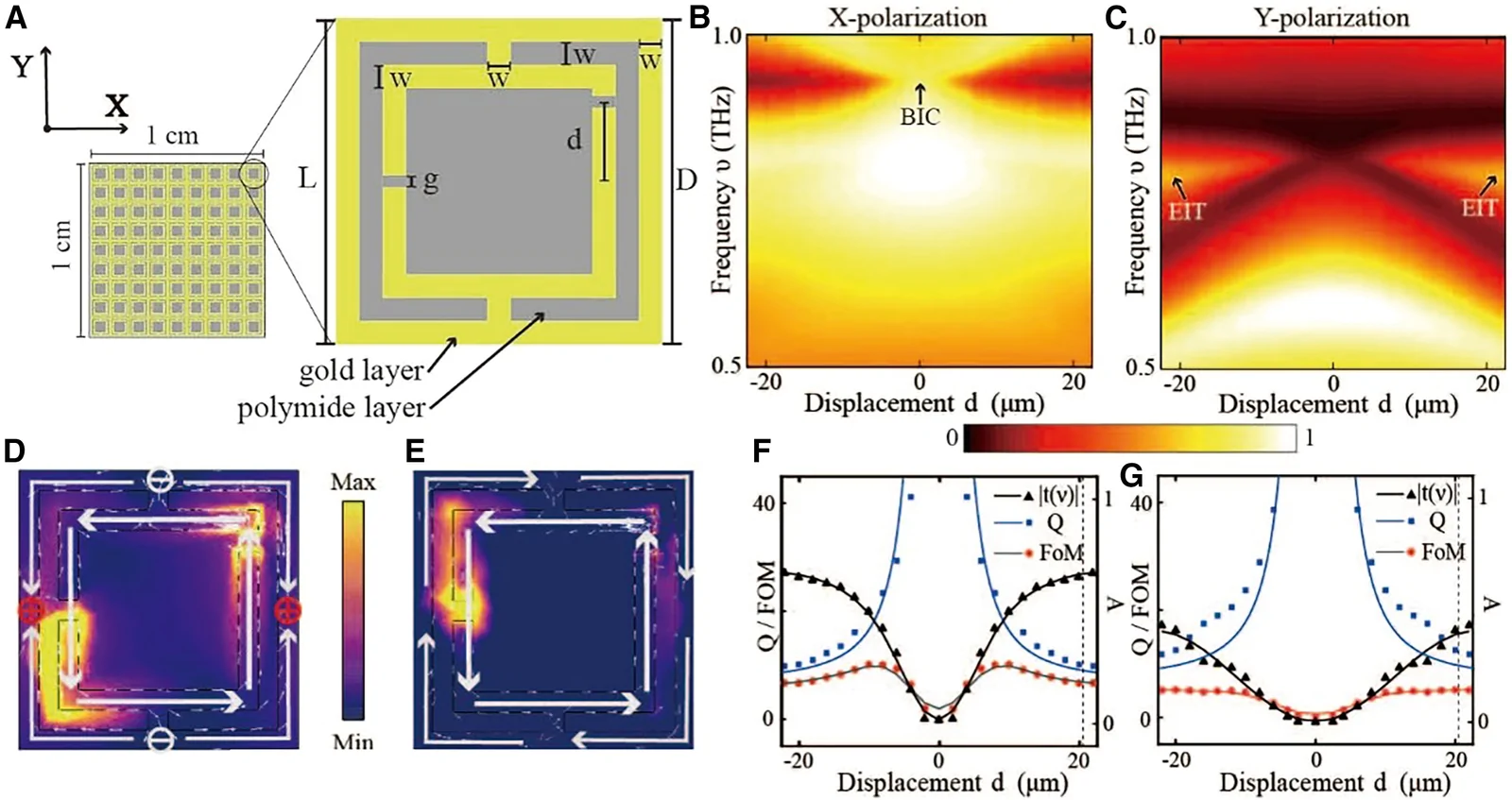
Structure and resonances of the THz metasurface (A) The unit-cell of the metasurface-based THz biosensor. *L*, length; *D*, unit cell length; *w*, width; *g*, gap; *d*, displacement. (B and C) The simulated transmission amplitude as a function of frequency and *d* excited by (B) *E*_*x*_, and (C) *E*_*y*_ incident field. (D and E) The distribution of total absolute field and surface current excited by *E*_*x*_ at the q-BIC frequency (see black arrow in B, left), and (E) excited by *E*_*y*_ at the EIT (see black arrows in C, right). (F) Resonance amplitude (|*t*(*ν*)|), *Q* factor (*Q*), and figure-of-merit (*FoM*) of the Fano-resonance line-shaped quasi-BIC as a function of *d* excited by *E*_*x*_. (G) |*t*(*ν*)|, *Q*, and *FoM* of the EIT as a function of *d* excited by *E*_*y*_.

**Figure 3:**
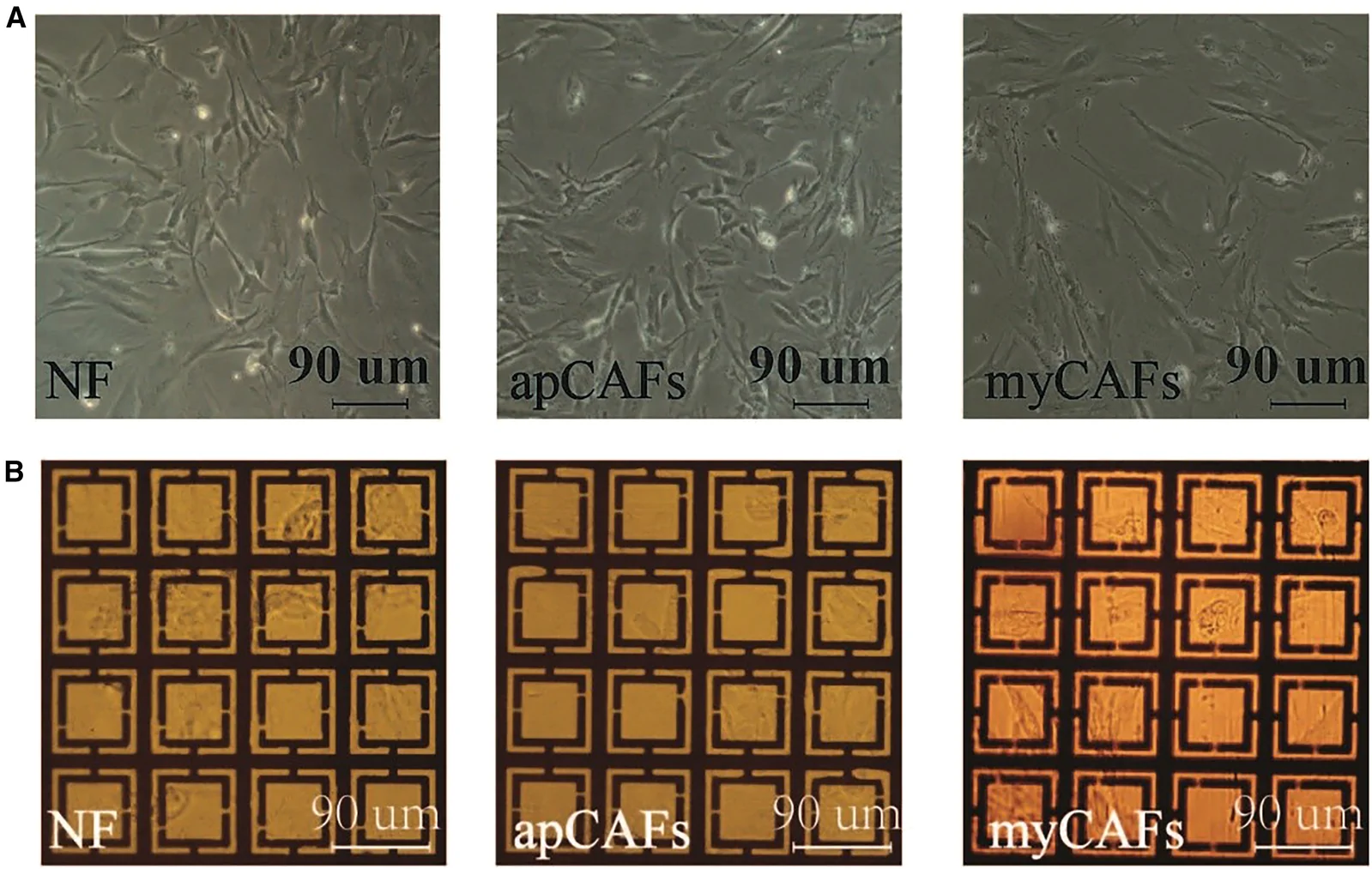
Deposition of fibroblast onto the THz metasurface (A) Optical microscopy images of normal intestinal fibroblast (NF), myofibroblast (myCAFs), and antigen-presenting CAFs (apCAFs) cell subtypes, respectively. (B) Optical microscopy images of macrophages from NF, myCAF, and apCAF subtypes deposited on the THz metasurface biosensor.

**Figure 4:**
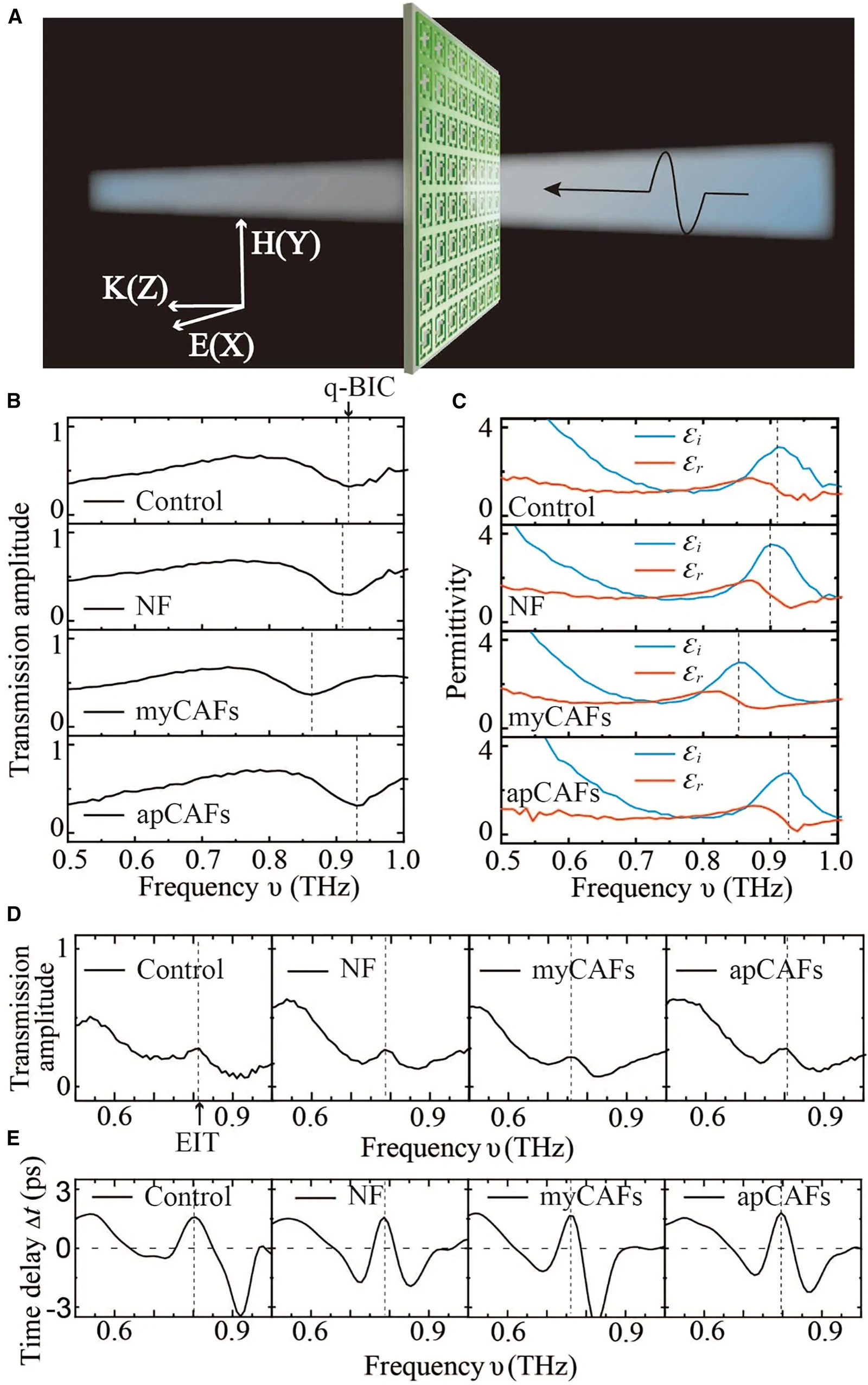
Characterization of the metasurface biosensors via THz-TDS (A) A schematic diagram of THz wave interaction with cells on metasurface-based biosensor. (B) THz spectral transmittance of analytes excited by *E*_*x*_. (C) The extracted permittivity of analytes. Here, the real (blue solid line) and imaginary (red) part of permittivity. (D and E) THz spectral transmittance (E) and the group delay dispersion of analytes excited by *E*_*y*_ (E).

As shown in Figure 4B, the transmission spectra of the chemically functionalized metasurface excited by *E*_*x*_ are plotted as a function of frequency for the control (without immobilized cells) and different CAF subtypes (NF, myCAFs, and apCAFs). All spectra exhibit resonant features within the 0.5–1.0 THz range.

Clearly, the resonant spectral profiles of different CAF subtypes display distinct asymmetric quasi-bound state in the continuum (q-BIC) resonance modes. The corresponding parameters of Fano-resonance lineshaped q-BIC are listed in Table 1.

**Table 1:** The parameters for Fano-resonance line-shaped q-BIC

Analytes	*ν*_0_ (THz)	*γ* (THz)	*Q*
Control	0.92	0.10	9.2
NF	0.91	0.09	10.1
myCAFs	0.86	0.10	8.6
apCAFs	0.94	0.10	9.2

Specifically, the undifferentiated healthy normal intestinal fibroblast (NF) sample exhibits a q-BIC resonance at 0.91 THz, showing a 10 GHz redshift in the central frequency compared to the control. When the metasurfaces are coated with pro-tumorigenic myCAFs, the introduction of these fibroblasts alters the surface dielectric environment (refractive index in the proximity) of the asymmetric double split-ring resonator (ADSRR), resulting in a more pronounced redshift of 60 GHz down to 0.86 THz. In contrast, the metasurfaces coated with anti-tumorigenic apCAFs experience a blueshift of 20 GHz up to 0.94 THz. The Fano-resonance linewidth (*γ*) of analytes are all about 0.1 THz, and the corresponding *Q*-factors are approximately 9,which are indistinguishable to different CAFs.

Such frequency shifts can be primarily attributed to changes in the local dielectric environment surrounding the metasurface, induced by the different CAF subtypes. Based on perturbation theory for resonant cavities, the frequency shift (<*ν*> of resonance modes induced by a localized change in permittivity (Δ*ε*(*R*,*ν*)) is typically proportional to the integral of the squared electric field intensity over the volume where the change occurs^42^:

(Equation 1) $$<\nu >\propto -2\pi \nu {∭}_{V}\Delta \varepsilon \left(R,\nu \right)E^{2}\left(r\right)d^{3}r\text{.}$$

Here, the functions of the complex permittivity at THz frequencies are listed as^43^

(Equation 2) $$\varepsilon \left(\nu \right)=\varepsilon_{r}\left(\nu \right)+i\varepsilon_{i}\left(\nu \right)\text{,}$$

where subscripts *r* and *i* represent the real part and imaginary part of permittivity, respectively. The dielectric functions are extracted from the THz transmission spectra presented in Figure 4B and are illustrated in Figure 4C.

Since CAFs and their surface hall-markers are non-magnetic materials, changes in permeability within the metasurface can be neglected. According to the effective medium theory, the dielectric constant (*ε*(*ν*)) of the cell layer is proportional to the cell number and types. In our study, the surface densities of the three CAF subtypes—NF, myCAFs, and apCAFs—are fixed at the same level of 10^6^ units/mL. Therefore, the influence of cell numbers to the frequency shift is negligible. Consequently, the biochemical properties of the CAF subtypes are considered as the variation in the dielectric environment of the metasurface, resulting in the frequency shift of the quasi-BIC resonances.

Figure 4D presents the EIT spectra of the metasurface excited by *E*_*y*_ THz wave for the control sample (without cells) and different CAFs (NF, myCAFs, and apCAFs) in the frequency range from 0.5 to 1.0 THz. The corresponding parameters of EIT resonances are listed in Table 2.

**Table 2:** The parameters for EIT

Analytes	*ν*_0_ (THz)	Δ*τ* (ps)
Control	0.82	1.58
NF	0.79	1.57
myCAFs	0.76	1.65
apCAFs	0.81	1.80

Obviously, the healthy normal fibroblasts (NFs) exhibit an EIT window at 0.79 THz, which shows a 30 GHz redshift compared to the resonance of the control sample. Regarding the NF as a reference, the pro-tumorigenic myCAF-coated biosensor induces a frequency redshift of 30 GHz, down to 0.76 THz. In contrast, functionalization with anti-tumorigenic apCAFs results in a blueshift of 20 GHz up to 0.81 THz. Compared to Figure 4B, the frequency shift trend of EIT is consistent with the q-BIC behavior observed excited by *E*_*x*_. Another important criterion of the EIT modes is the group delay (Δ*τ*) within the spectral transparency window, which is illustrated as the group delay of THz wave-packet propagating through the medium. This key parameter can be derived from the phase of the transmission spectrum^35^:

(Equation 3) $$\Delta \tau =-\frac{1}{2\pi }\frac{d\varphi \left(t\right)}{df}\text{,}$$

where *φ*(*t*) and *f* refer to the transmission phase and the frequency, respectively. Figure 4E displays the group delay as a function of frequency *f*, for different CAFs (NF, myCAFs, and apCAFs) in experiments. The THz spectra of Δ*τ* are in consistent with the spectral shift of EIT windows described in Table 2. The myCAFs increase the Δ*τ* from 1.57 up to 1.65 ps, while the apCAFs increases the Δ*τ* from 1.57 up to 1.80 ps compared to the healthy NF. Upon to the optics principle, the dispersion at the EIT windows determines the Δ*τ* of incident THz wave-packet.^44^ It seems that different CAFs have the slightly different dispersion ratio at the EIT window.

Supported by our recent findings,^22^ the dielectric function of cell is based on the surface hall-marks. The colorectal tumor proliferation or regression can be monitored by the ratio of myCAFS and apCAFs. Because abovementioned two types of CAFs express different levels of genes and key-proteins on their surface hall-marks of cells, including cytokines, chemokines, and other signaling molecules. Generally, myCAFs express α-SMA, PDPN, COL1A1, FAP, IL-6, TGF-β, and VEGF, at high level as well as chemokines, such as COL8A1, COL10A1, COL11A1, and COL12A1.^9^^,^^45^ In contrast, apCAFs highly express CD74, PDPN, VEGF, IL-6, and chemokines including CXCL13, CCL3, CCL4, and CCL5.^9^^,^^45^ Importantly, the full genetic regulatory mechanisms and signaling pathways remains unclear until today. Nevertheless, aforementioned expression patterns of different CAF subtypes either promote or inhibit tumor growth and metastasis, which dominate their extracellular matrix so that they have influence on the surface dielectric environment of the CAFs. Therefore, the subtly dielectric variation leads to the frequency shift of q-BIC and EIT of THz metasurface biosensor.

To confirm the different expression routes mentioned earlier, PDPN and CD74 are selected as hall-markers to distinguish undifferentiated intestinal epithelial cells from myCAF and apCAF subtypes. Figure 5A shows the relative gene expression levels of PDPN and CD74. Their corresponding protein expression levels are presented in Figure 5B.

**Figure 5:**
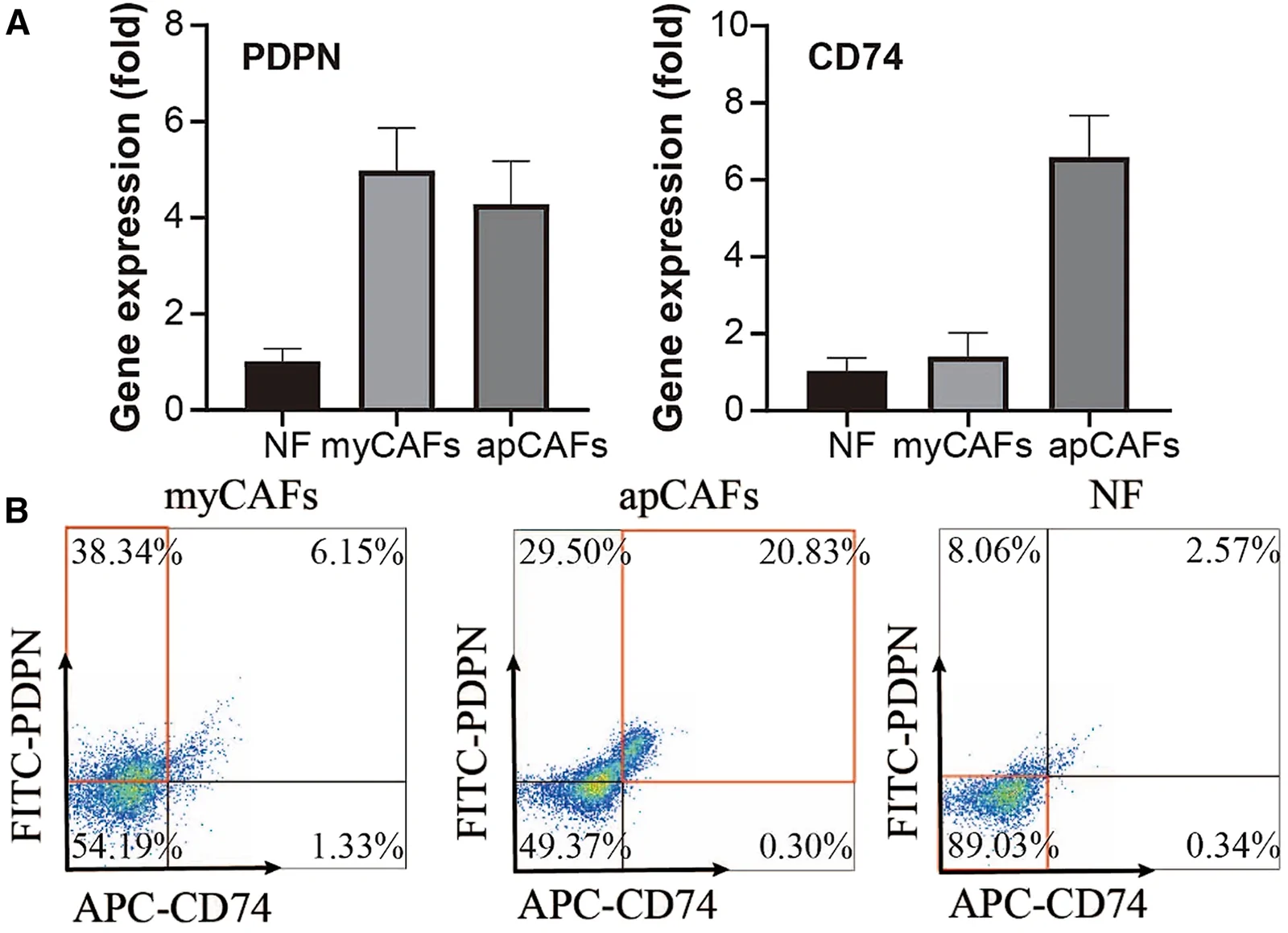
Surface hallmark of CAFs (A) Gene expression of hallmarks CD 74 and PDPN. (B) The measurement results by flow cytometry of NF, myCAFs, and apCAFs subtypes, respectively.

Cells expressing double-positive for both CD74 and PDPN are identified as apCAFs, which promote T cell activation and enhance their cytotoxicity and proliferative capacity, thereby strengthening T cell-mediated anti-tumor immunity. In contrast, cells negative for CD74 but positive for PDPN are classified as myCAFs. In normal intestinal epithelial tissue, healthy NFs express dual negative for both PDPN and CD74. Such a difference of surface hall-marks means that myCAFs and apCAFs have differential extracellular matrix, which comprises a wide variety of macromolecules with diverse functions, including collagens, elastin, laminin, and a variety of proteoglycans.^45^^,^^46^ Owing to the varied ratio of aforementioned macromolecules, the dielectric constant of different subtypes of CAFs is slightly different, leading to the frequency shift of q-BIC and EIT windows. It is important to note that quantifying the exact impact of these macromolecules on the extracellular matrix of each CAF type remains a complex challenge. In summary, we examined how contactless and contact interactions between adjacent meta-atoms affect SP-BIC. Our findings show that SP-BIC appears at 0.9 THz when meta-atoms are stimulated by *E*_*x*_ terahertz waves under both interaction conditions. When meta-atoms are excited by *E*_*y*_ terahertz waves, the frequency of the individual SP-BIC shifts to 0.8 THz without contact, while contact interaction transforms the SP-BIC into an EIT window at 0.8 THz, resulting in a measured group delay of 3.6 ps for slow light. Temporal coupled mode theory was used to clarify the underlying physical mechanisms, and the distribution of surface current along with multipole expansion helped explain the origins of quasi-BIC in both types of interactions. Numerical simulations reveal that the electric dipole primarily governs the quasi-BIC response for meta-atoms excited by *E*_*x*_ and *E*_*y*_ terahertz waves under contactless conditions. For contact meta-atoms, the quasi-BIC response remains similar to that of contactless meta-atoms excited by *E*_*x*_ waves, but with *E*_*y*_ waves, two oppositely polarized magnetic dipoles produce the EIT window in the contact interaction case. These results highlight the effects of contactless and contact interactions on the quasi-BIC in terahertz metasurfaces, which is important for advancing terahertz biosensor technology.

In summary, we examined how contactless and contact interactions between adjacent meta-atoms affect SP-BIC. Our findings show that SP-BIC appears at 0.9 THz when meta-atoms are stimulated by *E*_*x*_ terahertz waves under both interaction conditions. When meta-atoms are excited by *E*_*y*_ terahertz waves, the frequency of the individual SP-BIC shifts to 0.8 THz without contact, while contact interaction transforms the SP-BIC into an EIT window at 0.8 THz, resulting in a measured group delay of 3.6 ps for slow light. Temporal coupled mode theory was used to clarify the underlying physical mechanisms, and the distribution of surface current along with multipole expansion helped explain the origins of quasi-BIC in both types of interactions. Numerical simulations reveal that the electric dipole primarily governs the quasi-BIC response for meta-atoms excited by *E*_*x*_ and *E*_*y*_ terahertz waves under contactless conditions. For contact meta-atoms, the quasi-BIC response remains similar to that of contactless meta-atoms excited by *E*_*x*_ waves, but with *E*_*y*_ waves, two oppositely polarized magnetic dipoles produce the EIT window in the contact interaction case. These results highlight the effects of contactless and contact interactions on the quasi-BIC in terahertz metasurfaces, which is important for advancing terahertz biosensor technology.

### Limitations of the study

This study proposes a metasurface architecture that integrates both q-BIC and EIT effects for the identification and differentiation of CAF subtypes, which holds significant implications for clinical oncology of CRC. The experiments were conducted under ambient conditions using a THz-TDS system, with each CAF subtype being isolated, characterized, and cultured to a concentration of 10^6^ cells/mL prior to measurement.

First, from a physical perspective, due to the limited spectral resolution of the THz-TDS system, the frequency shift data obtained for some CAF subtypes are closely spaced. Achieving more distinct differentiation may require higher-precision instrumentation or higher cell concentrations. Second, from a biological perspective, human tumor tissues contain multiple CAF subtypes with considerable heterogeneity in concentration. Under such complex biological conditions, it remains challenging to directly isolate and identify specific CAF subtypes from patient-derived tissues. Finally, from a clinical perspective, the experimental conditions employed in this study—including cell concentration and biological environment—remain relatively idealized. Further experimental validation, together with the collection and analysis of pathological datasets, is essential to improve diagnostic accuracy. Consequently, substantial efforts are still required before this approach can be translated into routine clinical detection.

## Resource availability

### Lead contact

Requests for further information and code and data should be directed to and will be fulfilled by the lead contact, Zhenyu Zhao (zyzhao@shnu.edu.cn).

### Materials availability

This study did not generate new unique reagents.

### Data and code availability

• Data generated or analyzed during this study are publicly available.
• This paper does not report original code.
• Any additional information required to reanalyze the data reported in this paper is available from the lead contact upon request.

## Acknowledgments

This work was supported by the 10.13039/501100001809National Natural Science Foundation of China (no. 62475159). A.X. acknowledges support by the 10.13039/501100001711Swiss National Science Foundation (project INC-META no. 10.004.197); NTNU Rector AVIT fund (no. 949024104); and NTNU NanoLab via the Research Council of Norway support to the Norwegian Micro- and Nano-Fabrication Facility, NorFab (no. 295864). H.S.N acknowledges support by the French National Research Agency (ANR) under projects POLAROID (ANR-24-CE24-7616-01), SUPER-HERO (ANR-25-CE24-4066), and CHOCOLAT (ANR-44225-CE47-6432).

## Author contributions

H.X., writing – original draft, methodology, investigation, and data curation; R.G., methodology and data curation; A.G.S.T., methodology and data curation; I.G., methodology and data curation; A.X., supervision and writing – review and editing; Z.Y. and H.S.N., supervision and resources; Z.Z., supervision, writing – review and editing, and resources. H.X., R.G., A.G.S.T., and I.G. contributed equally to this work.

## Declaration of interests

The authors have no conflicts to disclose.

## STAR★Methods

### Key resources table

**Table:** 

REAGENT or RESOURCE	SOURCE	IDENTIFIER
**Antibodies**		

FITC anti-Human Podoplanin	BioLegend	Cat# 337025; RRID: AB_2750285
APC anti-human CD74	BioLegend	Cat# 326812; RRID: AB_2564389
Zombie Violet™ Fixable Viability Kit	BioLegend	Cat# 423113

**Oligonucleotides**		

CD74 qPCR forward primer: GACGAGAACGGCAACTATCTG	Tsingke	NA
CD74 qPCR reverse primer: GTTGGGGAAGACACACCAGC	Tsingke	NA
PDPN qPCR forward primer: AACCAGCGAAGACCGCTATAA	Tsingke	NA
PDPN qPCR reverse primer: CGAATGCCTGTTACACTGTTGA	Tsingke	NA

**Software and algorithms**		

FlowJo	NA	NA
GraphPad Prism 7	NA	NA
QuantStudio™ Real-Time PCR Software	NA	NA

**Other**		

FastPure Cell/Tissue Total RNA Isolation Kit V2	Vazyme	RC112-01
HiScript III RT SuperMix	Vazyme	R323-01
Taq Pro Universal SYBR qPCR Master Mix	Vazyme	Q712-02
Tissue Preservation Solution	Miltenyi Biotec	130–100–008

### Experimental model and study participant details

#### Human colorectal cancer and normal tissue samples

The myCAFs and apCAFs used in this study were isolated from surgically resected tumor tissues of patients with colorectal cancer (*n* = 3), while NFs were isolated from matched adjacent colonic tissues from the same patients. The study protocol was conducted in accordance with the Declaration of Helsinki and was approved by the Clinical Research Ethics Committee of Fudan University Shanghai Cancer Center (approval no. 050432-4-1911D). Written informed consent was obtained from all patients. Demographic information, including sex, age, and ethnicity, is provided in Table S1.

#### Cell culture

Human myCAFs, apCAFs, and NFs were cultured in DMEM/F-12 medium (Gibco, Cat. No. 11320033) supplemented with 10% fetal bovine serum (FBS; Gibco, Cat. No. A5669701) and 20 ng/mL basic fibroblast growth factor (bFGF; MedChemExpress, Cat. No. HY-P7004). Cells were maintained at 37°C in a humidified incubator containing 5% CO_2_. Cell identity was confirmed by the robust expression of fibroblast-specific markers. The cells were routinely verified as mycoplasma-negative.

### Method details

#### Biosensor preparation

Simultaneous THz q-BIC and EIT are realized with a metasuface of contact coupled asymmetric double-arm split-ring resonator (ADSRR),^47^ forming the basis of our THz biosensor. Figure 2A displays the fabricated metasurface sample with a total area of 1 cm^2^ with a periodicity *D* of 84 μm. The ADSRR is composed of an outer square ring connected via two small arms to an inner square structure. Two arms of width *g* = 3 μm are incorporated on the left and right sides of the inner square: one at the center of the left side and the other offset from the midline of the right side by *d* = 20.5 μm. No arm is introduced on the outer square. The line width of both the inner and outer squares is *w* = 6 μm, which is equal to the displacement between the two squares. The side length of the outer square is *L* = 84 μm, identical to the lattice constant *D*, while that of the inner square is 60 μm. The ADSRR structure was fabricated by depositing a 115 nm thick gold layer on a 25 μm thick polyimide (PI) film. The permittivity of the polyimide is 3.65. The permittivity of the polyimide used is 3.65 at 1 THz. Gold (Au) is modeled as a lossy metal with a conductivity of 4.56 × 10^7^ S·m^−1^. The permeability of all the materials is set as 1. The simulated results are obtained using a time-domain solver with commercial software CST Microwave Studio™. The unit-cell boundary conditions are applied in the *x*-*y* plane of meta-atom. The *z*-direction is open to free space. The input port and output port along the *z*-direction are set at 150 μm from the metasurface. The mesh density is set at 40 lines per wavelength. The simulated THz transmission spectra maps (as function of dispacement, *d*, and magnitude) under *x*-and-*y* polarization conditions are shown in Figures 2B and 2C. A symmetry-protected BIC is observed near 0.92 THz excited by *x*-polarized terahertz wave *E*_*x*_. Two EIT windows appear around 0.82 THz when the metasurface is excited by *y*-polarized THz wave (*E*_*y*_).

Further, as shown in Figure 2D, for the contact coupled meta-atom under *E*_*x*_ excitation, the surface current in the outer square flows toward the middle of the vertical arms, forming four mirror-symmetric L-shaped current paths. Each L-shaped path corresponds to an electric dipole oscillation. Meanwhile, the surface currents in the inner square circulate counterclockwise, indicative of a magnetic dipole resonance. According to the right-hand rule, the magnetic dipole moment is oriented along the wave-vector of incident THz wave. Thus, the quasi-BIC resonance under *E*_*x*_ excitation is hybrid of a magnetic dipole resonance coupled with the four L-shaped electric dipoles. Such an interference between the magnetic dipole resonance and four L-shaped electric dipoles resonant scattering process produces Fano-resonance q-BIC. Additionally, Figure 2E shows that the surface current on the outer square of the meta-atom flows clockwise, while the inner square’s current circulates counterclockwise, when the incident terahertz polarization in *E*_*y*_. These opposing current loops results in destructive interference of the magnetic dipoles, which manifests as an EIT window. It is noted that the detection sensitivity of THz biosensor attributes to the resonance amplitude (|*t*(*ν*)|, defined as the transmission amplitude at the q-BIC or EIT resonance), the quality factor (*Q*, defined as *Q* = *f*_0_/Δ*f*, where *f*_0_ is the resonant frequency and Δ*f* is the full width at half maximum), and the figure-of-merit (*FoM*, defined as *FoM* = *Q* × |*t*(*ν*)|).^48^ Figures 2D and 2E shows the *Q*, |*t*(*ν*)|, and *FoM* as a function of right-side gap displacement (*d*). In order to balance q-BIC and EIT performance excited under *E*_*x*_ and *E*_*y*_ condition, the *d* = 20.5 μm is determined in the design of our meta-atoms.

The ADSRR structure was fabricated by depositing a 115 nm thick gold layer on a 25 μm thick polyimide (PI) film. A poly (methyl methacrylate) (PMMA) A9 sacrificial layer was spin-coated at 1000 rpm for 30 s (Laurell WS-650MZ-23NPPB spin coater) onto a supporting 4-inch silicon (Si) wafer. A cleaned 50 × 50 mm polyimide film was then laminated onto the PMMA/Si wafer. Subsequently, the polyimide/PMMA/Si stack was cleaned with isopropyl alcohol and deionized water, followed by dehydration baking at 60 °C for 2 min. The designed pattern was fabricated on the polyimide substrate using maskless lithography (MLA100 maskless aligner – Heidelberg Instruments) with SPR700–1.0 photoresist, which was spin-coated at 3000 rpm for 34 s. Development was carried out in a ma-D 332 developer. A 10 nm layer of titanium (Ti) followed by a 115 nm thick gold (Au) film was subsequently deposited by electron beam evaporation (PVD 200, KURT J. Lesker) at a deposition rate of 3 Å s^−1^ and 5 Å s^−1^, respectively. Finally, acetone was used to remove unexposed photoresist and residual metal layers (lift-off). The freestanding Au/PI metasurface was released by dissolving the PMMA sacrificial layer through immersion in acetone for over 2 h.

#### Culture and transfer of CAFs

To examine the sensitivity of the THz metasurface sensor, freshly resected colorectal cancer tissues and adjacent normal intestinal epithelial tissues are collected and thoroughly rinsed five times with PBS buffer containing 20% antibiotic-antimycotic solution. The samples are then mechanically minced into 1–2 mm^3^ fragments using ophthalmic scissors. Subsequently, the tissue fragments are digested with collagenase type IV at 37 °C under constant agitation at 200 rpm for approximately 1 h. The digested suspension was filtered through a 70 μm cell strainer, and the filtrate is centrifuged at 1500 rpm for 5 min. This step is repeated 2–3 times to thoroughly remove residual enzymes. The resulting cell suspension is then subjected to fluorescence-activated cell sorting (FACS) for phenotypic identification using the following antibody panel: APC-conjugated anti-CD74 (BioLegend, Cat# 326811) and FITC-conjugated anti-PDPN (BioLegend, Cat# 337025). Three distinct fibroblast subpopulations are isolated: pro-tumorigenic myofibroblastic CAFs (myCAFs), tumor-suppressive antigen-presenting CAFs (apCAFs), and undifferentiated normal intestinal fibroblasts (NFs) with no significant functional roles. Sorted cells are cultured in appropriate medium and adjusted to a uniform concentration of 10^6^ cells/mL. Their morphological characteristics are shown in Figure 3A. The THz metasurface biosensors are functionalized using NHS/EDC chemistry, and the three types of CAFs are separately immobilized onto three identical chemically modified metasurfaces via paraformaldehyde fixation. Optical microscopy images of the immobilized CAFs subtypes on the THz metasurfaces are presented in Figure 3B.

#### Characterization

Figure 4A illustrates the response of the metasurface biosensor within the THz frequency range. The incident terahertz pulse propagates along the *z* axis, with the metasurface in the *xy*-plane. Transmission spectra of the metasurface biosensor were measured using a fiber-coupled terahertz time-domain spectroscopy (THz-TDS) system (TERA K15, Menlo Systems GmbH). A time window of 100 ms was applied, corresponding to a frequency resolution of 10 GHz. All measurements were conducted under a pure nitrogen atmosphere to minimize the influence of water vapor. Each sample is measured three times to ensure reproducibility. The acquired time-domain signals are converted into frequency-domain data via Fast Fourier Transform (FFT). The corresponding transmission amplitude is extracted using the following equation^35^:

(Equation 4) $$\left|t\left(v\right)\right|=\left|E_{\text{analyte}}\left(v\right)/E_{\text{ref}}\left(v\right)\right|$$

Here, |*t*(*ν*)| denotes the frequency-dependent THz transmission coefficient. *E*_analyte_(*ν*) and *E*_ref_(*ν*) are defined as the THz electric fields transmitted through the analyte-loaded and the reference (unmodified) metasurface, respectively.

#### Simulation

The simulated results are obtained using a time-domain solver with commercial software CST Microwave StudioTM. The unit-cell boundary conditions are applied in the *x*-*y* plane of meta-atom. The *z*-direction is open to free space. The input port and output port along the *z*-direction are set at 150 μm from the metasurface. The mesh density is set at 40 lines per wavelength.

#### Fabrication

The ADSRR structure was fabricated by depositing a 115 nm thick gold layer on a 25 μm thick polyimide (PI) film. A poly(methyl methacrylate) (PMMA) A9 sacrificial layer was spin-coated at 1000 rpm for 30 s (Laurell WS-650MZ-23NPPB spin coater) onto a supporting 4-inch silicon (Si) wafer. A cleaned 50 × 50 mm polyimide film was then laminated onto the PMMA/Si wafer. Subsequently, the polyimide/PMMA/Si stack was cleaned with isopropyl alcohol and deionized water, followed by dehydration baking at 60 °C for 2 min. The designed pattern was fabricated on the polyimide substrate using maskless lithography (MLA100 maskless aligner – Heidelberg Instruments) with SPR700–1.0 photoresist, which was spin-coated at 3000 rpm for 34 s. Development was carried out in a ma-D 332 developer. A 10 nm layer of titanium (Ti) followed by a 115 nm thick gold (Au) film was subsequently deposited by electron beam evaporation (PVD 200, KURT J. Lesker) at a deposition rate of 3 Å s^−1^ and 5 Å s^−1^, respectively. Finally, acetone was used to remove unexposed photoresist and residual metal layers (lift-off). The freestanding Au/PI metasurface was released by dissolving the PMMA sacrificial layer through immersion in acetone for over 2 h.

#### Characterization

The incident terahertz pulse propagates along the *z* axis, with the metasurface in the *xy*-plane. Transmission spectra of the metasurface biosensor were measured using a fiber-coupled terahertz time-domain spectroscopy (THz-TDS) system (TERA K15, Menlo Systems GmbH). A time window of 100 ms was applied, corresponding to a frequency resolution of 10 GHz. All measurements were conducted under a pure nitrogen atmosphere to minimize the influence of water vapor.

#### Flow cytometric analysis and cell sorting

Fresh colorectal cancer tumor tissues were mechanically dissociated and enzymatically digested to generate single-cell suspensions as described above. Cell suspensions were filtered through a 40-μm cell strainer to remove debris and cell aggregates, washed with phosphate-buffered saline (PBS), and resuspended in PBS supplemented with 2% fetal bovine serum (FBS).

Cell viability was assessed using the Zombie Violet Fixable Viability Kit (BioLegend) according to the manufacturer’s instructions, and dead cells were excluded from subsequent analyses. For surface marker staining, cells were incubated with fluorophore-conjugated antibodies against human CD74 (BioLegend) and human podoplanin (BioLegend) for 30 min at 4°C in the dark. Following staining, cells were washed twice with PBS containing 2% FBS and resuspended for flow cytometric analysis and sorting. Based on the expression of PDPN and CD74, sorted fibroblast populations were classified as follows: PDPN^+^CD74^+^ cells were defined as antigen-presenting cancer-associated fibroblasts (apCAFs), PDPN^+^CD74^−^ cells as myofibroblastic cancer-associated fibroblasts (myCAFs), and CD74^−^PDPN^-^ cells as NFs.

Flow cytometric analysis and fluorescence-activated cell sorting were performed on a BD FACSAria III. Compensation was established using single-stained controls. Sorted cells were collected into tubes containing 1640 (RPMI-1640) and immediately processed for downstream applications. Flow cytometry data were analyzed using FlowJo.

#### Quantitative PCR

Total RNA was extracted from sorted cells using FastPure Cell/Tissue Total RNA Isolation Kit according to the manufacturer’s instructions. Complementary DNA (cDNA) was synthesized using HiScript III RT SuperMix for qPCR. Quantitative PCR (qPCR) was performed using gene-specific primers for CD74 and PDPN (listed in the key resources table) on a QuantStudio Real-Time PCR machine. Gene expression levels were normalized to ACTB, and relative expression was calculated using the 2ˆ−ΔΔCt method.

### Quantification and statistical analysis

Data presented in Figure 5A are shown as the mean ± standard deviation (SD). Statistical comparisons were performed using Student’s *t* test. Each group included three biological replicates. A *p* value <0.05 was considered statistically significant.

## Supplemental information

Table S1. Characteristics of patients included in the study
